# Fish Welfare in Public Aquariums and Zoological Collections

**DOI:** 10.3390/ani13162548

**Published:** 2023-08-08

**Authors:** Stephen A. Smith

**Affiliations:** Department of Biomedical Sciences and Pathobiology, Virginia Maryland College of Veterinary Medicine, 205 Duck Pond Drive, Virginia Tech, Blacksburg, VA 24061-0442, USA; stsmith7@vt.edu

**Keywords:** fish, husbandry, welfare, enrichment, assessment

## Abstract

**Simple Summary:**

Numerous species of fish are displayed in public aquariums and zoological collections all over the world. A general description of the basic biological requirements for maintaining fish in captive environments is presented, where a combination of behavioral, performance, and physiological indicators can be used to assess the welfare of these animals. Ultimately, the goal for optimizing fish welfare should be to provide the best possible environment, husbandry, and social interactions to promote natural species-specific behaviors of the fish in captivity.

**Abstract:**

A wide variety of fish species have been displayed in public aquariums and zoological collections for over 150 years. Though the issue of pain perception in fish is still being debated, there is no disagreement that negative impacts on their welfare can significantly affect their health and wellbeing. A general description of the basic biological requirements for maintaining fish in captive environments is presented, but species-specific information and guidelines should be developed for the multitude of species being maintained. A combination of behavioral, performance, and physiological indicators can be used to assess the well-being of these animals. Ultimately, the goal for optimizing the welfare of fish should be to provide the best possible environment, husbandry, and social interactions to promote natural species-specific behaviors of the fish in captivity.

## 1. Introduction

Ever since the first aquarium exhibit opened at the London Zoo in 1853, aquatic animals have been a mainstay of public aquariums and zoological collections [1]. Unfortunately, their care and welfare has not always kept pace with their terrestrial counterparts. This has gradually changed over the last 30+ years as aquarists have become more knowledgeable about their captive fish species and more veterinarians have become trained in aquatic animal medicine. Along with this has been an increased awareness of the welfare of fish maintained in captivity whether they be for home aquariums, public display, stock enhancement, or food production.

During the past 20 years, numerous monographs and guidelines have been published on the care and maintenance of laboratory/research fish [2,3,4,5,6,7,8,9,10,11,12]. The *Guide for the Care and Use of Laboratory Animals* also includes recommendations for the housing, care, and well-being of fish used in research [13], along with additional recommendations by Mason and Matthews [14]. Though most of these pertain specifically to selected laboratory species such as zebrafish (*Danio rerio*), Japanese medaka (*Oryzias latipes*), fathead minnows (*Pimephales promelas*), platyfish and swordtails (*Xiphophorus* spp.), guppies (*Poecilia reticulata*), killifish, and sticklebacks, and goldfish (*Carassius auratus*), they do provide an excellent basis for general care and maintenance of common fish species [15,16]. There are also a number of excellent guides on the care and breeding of selected freshwater tropical fish species and some marine species, such as discus, bettas, guppies, freshwater angelfish, platies and swordtails, cichlids, corydoras catfish, and clownfish which can be useful when setting up aquatic exhibits [17,18,19,20,21,22,23,24]. In addition, there are numerous guidelines for the general care and maintenance of common food fish species, such as common carp (*Cyprinus carpio*), salmonids (*Oncorhynchus* spp., *Salmo* spp. and *Salvelinus* spp.), channel catfish (*Ictalurus punctatus*), tilapia (*Oreochromis* spp., *Tilapia* spp., and *Sarotherodon* spp.), striped bass (*Morone saxatilis*), and pangasius catfish (*Pangasianodon hypophthalmus*) that can be consulted [25,26,27,28,29]. Unfortunately, there is a limited amount of species-specific information available for the majority of non-domesticated fish species displayed in most aquariums and zoological settings. Thus, ideally before any fish are obtained by a facility, a thorough literature search on the natural history, behavior, social interactions, and water quality and nutritional requirements should be undertaken.

## 2. Fish Welfare

An important prelude to understanding fish welfare is the continuing discussion over the past several decades as to whether fish have the ability to experience discomfort, distress, suffering, fear or pain. One side of the argument is that fish do not have the necessary anatomy or mental awareness to recognize pain [30,31,32], while the other side of the argument is that fish have the appropriate nociceptors, neural pathways, and central neural mass to perceive, recognize, and respond to pain [33,34,35,36,37,38,39]. Common to both sides is that fish have the nociceptive abilities and are able to reflexively respond to noxious stimuli, but the sides diverge over whether fish can suffer discomfort or experience pain as a conscious experience (i.e., sentience).

Recent scientific literature arguably supports the premise that fish have the necessary processes for being aware of negative welfare experiences. Briefly, these include the presence of specific anatomical receptors for noxious stimuli (i.e., touch, heat, and chemical), neurological tracts that contain both A-fibers (i.e., myelinated) and C- fibers (i.e., unmyelinated) for the conduction of nerve impulses, increased activity in the forebrain to noxious stimuli, the presence of opioid pathways including receptors and endogenous ligands, altered gene expression in the forebrain to prolonged stimulation of nociceptors, and subsequent changes in behavior to noxious stimuli [40,41,42]. Thus, fish are anatomically, molecularly, biochemically, pharmacologically, and behaviorally similar to higher vertebrates and, therefore, in all likelihood experience some form of adverse state in response to aversive or undesirable situations [33,43]. It has also been shown that fish can learn and remember negative experiences suggesting that fish have the cognitive and behavioral capacities to process various types of information and respond appropriately [33]. In addition, several recent studies in fish have demonstrated their ability to perceive and recognize a reflected mirror image (i.e., mirror self-recognition test) which in humans and other primates is interpreted as the capacity for self-awareness [44,45,46]. Thus, the veterinary and scientific community has the ethical responsibility to address factors that could negatively affect the welfare and wellbeing of fish in any collection maintained for public display, interactive encounters, or conservation breeding.

Similar to terrestrial animals, fish welfare is a difficult concept to define. Recognizing poor welfare for an animal or a population of animals is generally easy for most casual observers, but good welfare is often harder to characterize. Fish welfare can be described as “striving for a positive state of well-being that allows the fish to be able to exhibit natural behavioral, social and physiological characteristics” [12]. This definition incorporates the “five freedoms”: (1) Freedom from hunger (e.g., having an appropriate, nutritionally complete, and adequate amount of food provided with appropriate growth rates); (2) Freedom from discomfort (e.g., having a suitable environment with appropriate water quality, temperature, light intensity, and shelter); (3) Freedom from injury or disease (e.g., being regularly monitored for clinical signs of disease); (4) Freedom to express normal behavior (e.g., by providing sufficient space, appropriate social groupings, substrate, and visual barriers if appropriate); and (5) Freedom from distress (e.g., by reducing or eliminating stressful situations from the environment when appropriate such as poor water quality, overcrowding, or aggressive tank situations) [12,47].

A recent concept for addressing welfare in animals, including fish, is the incorporation of the concept of “allostasis”, which proposes that sentient individuals are not just passive observers in their environment but are instead active participants. Accepting the fact that even an optimal environment may contain stressors allows fish the freedom to adapt and learn from minor negative experiences, thereby improving their quality of life. For instance, low levels of stress may have a positive effect on competition and reproductive success, while greater levels of stress (i.e., distress) may result in deleterious effects on the performance and health of the fish [48,49]. However, adaptive responses do not appear to be equal in all species of fish, such as European seabass (*Dicentrarchus labrax*) which appear less resilient to stress compared to gilthead seabream (*Sparus aurata*) [50]. Thus, species specific physiological differences need to be considered when evaluating the ability of a species to adapt to the various challenges of a particular environment [50].

There have been a number of books and numerous journal publications on fish welfare in general and on specific aspects of fish welfare including descriptions of welfare considerations for both laboratory-maintained species and aquaculture species [12,51,52,53]. Unfortunately, there has been very little published on the welfare of fish species in public aquarium displays and interactive encounters though this has recently been changing [54,55]. Considering the extreme diversity of fish species being maintained in captivity for public display or interactive encounters, no standard set of welfare criteria will work for all species, and welfare assessments must be tailored to each specific species or different groups of species in an exhibit. As a result, it should be understood that not all display tanks will be handled the same way, e.g., different water qualities, different temperatures, different lighting, different species, different diets, population/stocking density, social grouping, and so forth. Therefore, what follows are general welfare considerations for fish maintained in aquariums and zoological collections.

## 3. Water Quality

The importance of water quality in fish exhibits and habitats cannot be overstated; water composition is essential to the health and wellbeing of all fish species [13]. However, different species and life stages may require similar or extremely different water quality parameters to provide appropriate environmental conditions for optimal health and welfare. Obviously marine, brackish, and freshwater species generally require different salinities. While many species will adjust to variations close to their optimal salinity, some species may require certain salinities depending on life stage or reproductive cycle (e.g., anadromous vs. catadromous). Knowing the basic biology of the species being maintained is extremely important, and may dictate that some species cannot be maintained together. For instance, keeping channel catfish (i.e., a warm water species) together with Arctic char (i.e., a cold water species) at either species’ preferred temperature would not be the optimal environment for one of the species. Other water quality parameters are often less subtle in their effects on fish. For example, freshwater angelfish will generally not spawn in hard water, while striped bass fry require hard water to develop properly. Therefore, acquiring as much information about the natural aquatic environment for each of the species being maintained is essential for their wellbeing. Water quality monitoring at a minimum should include parameters such as temperature, pH, ammonia, nitrite, nitrate, hardness, alkalinity, dissolved oxygen, and chlorine/chloramines for all species. However, depending on the specific-species life support system (i.e., mechanical and biological filtration, circulation, aeration, clarification, and disinfection), and water source, other parameters such as salinity, copper, phosphorous, ions and minerals, total gas saturation, conductivity, and oxidation–reduction potential may be required as necessary [14]. Regular monitoring of water quality parameters and permanent records of those parameters can provide useful information for stabilizing and optimizing an aquatic environment for fish.

Water quality parameters outside the tolerant range of the species can cause significant changes in a number of physiological and biochemical properties of a fish resulting in abnormalities of blood chemistry, hematology, changes in the cellular permeability, and changes in the osmoregulatory needs of the fish. The resulting “stress response”, or disturbance in the balance of the physiological homeostasis, initiates a variety of adaptive responses to help return the fish to a stable homeostatic state [56,57]. Failure of these adaptive mechanisms to return to a normal physiological state produces an aversive negative state that can result in decreased wellbeing of the fish. This can ultimately manifest as observable changes in behavior and/or external skin and gill lesions depending on the type, magnitude, and acute or chronic nature of the stressor. These observable deviations from normal are generally considered a tertiary (i.e., whole animal) response to stress, which means the fish has already gone through the primary (i.e., hormonal blood changes) and secondary (i.e., cell and tissue changes) stress responses trying to adapt to the environment insult. Thus, by the time there are observable behavioral or clinical signs, the fish is already significantly compromised making welfare intervention challenging.

## 4. Species Selection

Selection of the species of fish to use for display or interactions depends on a variety of factors, including the type of exhibit, aquatic space available, life support system constraints, husbandry requirements, social compatibility, collectability, tolerance to captivity and handling, and disease susceptibility/resistance. Historically, most fish have been collected from the wild and maintained in aquaria on a trial-and-error basis with evaluation of morbidity and mortality attributable to collection, shipping, handling, species compatibility, water quality, and diets. Today, with the substantial historical perspectives of aquariums and zoos, advancement in scientific literature and communication between facilities, most commonly housed and novel species have a reasonable chance of survival and even thriving in the captive environment.

The compatibility of multiple species within the same exhibit should also be given serious consideration. Themed exhibits based on geographic locations often include a diverse community of various species including high end predators and lower prey items. However, it is not desirable to depend on the predator–prey relationship that would be found in nature for feeding individuals in the exhibit as this would present a poor welfare situation for the prey species. Thus, we must find ways to reduce the link between the predator and prey by selectively feeding the predators close to satiation, providing shelters/hides for prey species, and giving all species adequate space to avoid interaction with more dominant species. In addition, consideration should be given to gender selection, if possible, as intraspecific sexual and territorial aggression can cause significant behavioral disturbance in a community tank.

## 5. Nutrition

The specific nutritional requirements are not known for the majority of fish species kept in captivity. Although formulated feeds for aquaculture species such as carp, salmonids, and catfish are commercially available, these are often inappropriate for the diverse species of fish maintained in public aquaria. Thus, the general recommendation is to feed a palatable diet that meets the daily nutritional and behavior requirements of the species [13]. This should involve investigating the natural history of the species to determine if the fish is a carnivore, herbivore, or omnivore so that the feed ingredients can be tailored to the species. Not all fish species will readily accept a pelleted, flake, or gel diet and often need to be acclimated separately from an established population to ensure they are eating an adequate amount of food before being mixed with other species. Some aquariums feed more natural diets such as whole dead fish or fillets (e.g., mackerel, herring, capelin, and trout), shrimp, squid, molluscs, and other frozen or live organisms (e.g., blood worms, krill, brine shrimp, and algae) but need to make sure these items are contaminant-free and pathogen-free. If the fish species does not recognize or accept the offered diet, the food will generally decompose at the bottom of the tank causing water quality problems or be eaten by other species of fish in the tank leading to unintended obesity, either of which can result in a negative welfare situation. In addition, some species of fish will feed only once a day or only once a week, while other fish are natural grazers that feed continuously [58,59]. Finding a balance between natural behavior, feeding, and water quality is essential for a healthy environment and fish population.

## 6. Habitat Design

Any physical habitat for fish should be made of materials that are either nontoxic or coated with a material that is nontoxic for the fish. The interior of the system should be free of sharp edges, abrasive surfaces, and hard to access spaces for aquarium personnel. The space necessary for a particular species or mixed group of species will generally determine the size, volume, and depth of the habitat. The requirements for normal postural positioning by the fish in the exhibit and adjustments for increases or decreases in population densities need to be taken into consideration. Intake and discharge pipes should have size appropriate covers to prevent entrapment or fish from escaping the enclosure and getting into the recirculation systems (i.e., sumps and pumps). As an extra precaution, all systems should have traps or baskets that are regularly monitored to catch any escapees before entering the recirculation systems.

The substrate should be of material that is safe, nontoxic, and pathogen-free, such as sterilized sand, gravel, or crushed coral. Substrates play an important role in the behavior and welfare of fish, and studies have shown a particular preference by species and even individuals within a species. For instance, corydoras catfish (*Corydoras* spp.) have a preference for sand over larger gravel to facilitate their normal foraging behavior. It has also been shown that Nile tilapia have individual preferences for different sizes of substrate [60]. If no substrate is provided, the color of the bottom and sides of the habitat may be important for larval and postlarval fish, as well as a less stressful environment in many adult species [61,62,63]. For instance, juvenile turbot (*Scophthalmus maximus*) had higher growth rates, feed intake, and metabolic rates (including oxygen consumption rate, and ammonia excretion rate) with blue and white backgrounds, and lower levels with black, red, and brown backgrounds [64]. Similarly, the lined seahorse (*Hippocampus erectus*) showed a preference for white and blue backgrounds over black and red backgrounds [63]. In addition, shelters, hides, and shaded areas can be provided to allow fish safe areas for protection or to remove themselves visually from other inhabitants of the enclosure [65].

Controls for temperature and lighting should allow for seasonal changes or to induce breeding if desired. Additionally, light controls should be advanced enough to allow for dusk and dawn adjustments rather than lights snapping on and off which can startle fish and cause them unnecessary stress or sometimes even cause them to jump out of tanks. Remember that not all fish species are diurnal, and that some species avoid lighted areas of an exhibit. Again, a through literature search of the biology of a particular species should help determine the approximate preferred brightness (i.e., lumens) and temperature (i.e., kelvin) of light in nature.

## 7. Enrichment

Enrichment is an animal welfare principle that endeavors to enhance the quality of life for captive animals by identifying, providing, and assessing environmental stimuli encouraging optimal psychological and physiological well-being [Y]. Enrichment can be either active or passive, and is intended to stimulate behavioral outcomes similar to the species’ natural history [66]. Enrichment activities for fish should be used whenever possible to make the captive environment more stimulating and allow the fish to express natural species-appropriate behaviors. However, due to the wide diversity of fish species in public displays with varying natural histories and different physiological and behavioral traits, a species-specific approach for the various species may be required to adequately provide appropriate enrichment [12,67,68,69,70]. Examples of enrichment for aquarium fish may include changes in the environment, diet, or social interaction of the fish [71]. Alterations of the environment can include providing structures or shelters in the environment for cover or visual barriers, changes in background color, changes in water flow direction in the system, periodic changes in the photoperiod or intensity of lighting, and moving items or adding novel items in the aquarium [12]. The addition of a suitable substrate in the aquatic system may be used to encourage species-appropriate activities such as foraging. Dietary enrichment can include feeding of different types of food, feeding at different times of day, or feeding in a different location in the aquarium system. Creating different types of feeders or challenges for feeding could also be an enrichment for a particular species. Activities that encourage species-specific social interaction should be incorporated whenever possible, such as having enough fish of the same species (e.g., neon tetras, *Paracheirodon innesi*; glass catfish, *Kryptopterus vitreolus*; bluestriped grunts, *Haemulon sciuru*; and lookdowns, *Selene vomer*) to encourage schooling or shoaling activity if that is a normal part of their natural behavior. As this activity has been theorized to be for protection or feeding behavior, having only one or two individuals of a schooling or shoaling species in an aquarium system would not be a positive welfare situation for those fish. Conditioning or training, such as target feeding, of fish can be used to selectively feed specific individuals or used to accomplish certain tasks, and should also be considered an enrichment activity. However, providing what is initially perceived as enrichment does not always result in positive experiences for the fish. For instance, providing too few shelters for the number of fish in the exhibit may cause an increase in aggressive behavior in the population of fish due to competition for the shelter. Therefore, any enrichment should be monitored and evaluated for its goal and utility to provide a positive experience for the fish or population of fish.

## 8. Environments of Particular Welfare Concern for Fish

- **Interactive encounters in touch tanks**

The very nature of the public intimately interacting with captive aquatic organisms presents problems with conflicts of moral and ethical issues, in addition to failures in biosecurity, injuries, and potential exposure to toxins and pathogens, and, therefore, problems with fish welfare [72]. Fish can sometimes be exposed to unintentional or intentional inappropriate handling, substances on the hands of the public (e.g., sunscreen, lotions, soap, and other materials), and foreign objects entering the habitat. A species appropriate habitat with suitable substrate should be provided to optimize the environment. Exhibits for interactions should have water qualities carefully monitored more frequently than other exhibits, and water exchanges completed on an increased replacement cycle. An additional preventative measure that should be taken includes adding activated carbon filtration to the life support system to help remove unknown potentially toxic substances from the water.

Some fish species tolerate and even seek human interaction (e.g., koi and rays) either due to a desire for food or physical contact [73]. Interactive environments should always have areas in the aquarium where individuals can take refuge from the activity. However, some species or even individuals within a particular species that do not do well with public interactions would be best served to be removed from the interactive experience to reduce potential stress and preserve their welfare. Animals in an interactive exhibit should be monitored regularly for adverse reactions to the interaction, observed for signs of stress, poor health or abnormal behavior, and examined for indications of trauma or clinical signs of disease [74,75].

- **Quarantine**

Quarantine is an area where fish welfare is often overlooked due to generally more pressing needs such as the immediate health of the fish. Fish that are either recently acquired from the wild or individual(s) that are removed from their normal housing in the facility for health issues are usually put in an isolated tank that is completely unfamiliar. Obviously, it is extremely important to have a fully functional and biologically active filtration system working so that the fish has an optimal environment with the correct water quality, temperature, and dissolved oxygen for that species. However, most quarantine tanks are completely devoid of substrate, structures, shelters, and often other tank mates for ease of observation, treatments, and cleaning. Handling, lighting, change or acclimation to new diets, ambient noise or movement of people in the room where quarantine systems are placed may also cause additional stress in the fish. Individual(s) exposed to quarantine situations where the environment may be suboptimal for welfare may exacerbate the reason for the fish initially being in quarantine. Thus, whenever possible, the maintenance of fish in quarantine should include consideration of the welfare needs of these individuals.

## 9. Welfare Assessment

Practical assessment of animal welfare has historically used a model that utilizes three basic evaluation categories: (1) Behavioral-based indicators; (2) Functional-based indicators; (3) Physiological-based indicators (Table 1). The behavioral-based indicators are those traits that are used to visually observe and identify abnormal or maladaptive/sterotypic behaviors of a fish. These behaviors include submissive or aggressive activities, changes in food intake or foraging activities, and changes in schooling or shoaling activities [76,77]. Unfortunately, the majority of species of fish that are maintained in aquarium and zoological settings have never had a thorough documentation of their natural behaviors, and as a result the recognition of subtle abnormal or maladaptive behaviors becomes challenging and generally extrapolated from other species [78].

The functional-based category includes physical and/or performance-based indicators that are generally easy to observe or directly measured in a fish, such as physical abnormalities and production parameters. Physical indicators include lethargy, buoyancy problems, increased mucus production, weight loss, body abnormalities, increased respiratory rate, and skin and fin lesions, while production indicators include decreased growth rates, and decreased reproductive performance (e.g., egg production, hatchability, slow growth, and survival of juvenile fish). These fish health characteristics will vary depending on the species, age, and gender of the fish, and may also vary depending on the physical habitat and management of the exhibit.

Physiological-based indicators demonstrate alterations in the physiological, biochemical, or genetic makeup of the fish and generally require capture of the individual fish to take appropriate samples for laboratory evaluation. Parameters that are often measured include hematology and biochemistry, circulating levels of plasma cortisol, sex steroids, humoral antibodies, heat shock proteins, and the expression of genes. Again, this can be difficult to evaluate as standard values for most of the essential parameters are not known for most of the species maintained in public aquariums and zoological collections. In addition, the stress of capturing, handling, and sampling the fish can alter the desired values. However, advances have been made to develop noninvasive methods to evaluate some of these substances, such as cortisol, that are released into the water column [79,80,81,82]. There have also been several recent innovations in the field of biosensors for the assessment of fish health [83,84].

Incorporating a fourth category into this assessment model, that includes the basic environmental/management standards, is essential for an overall assessment of the fish or population of fish. This would require including the evaluation of parameters previously described in the discussion of water quality, nutrition, and habitat design into the overall welfare assessment. This is extremely important for example when dealing with extremes of water quality where fish may not show obvious clinical signs of detrimental water parameters for days to weeks or longer.

In addition to the three outcome-based system of welfare assessment, a relatively new and more robust model, based on the Five Freedoms and known as the “Five Domains”, has been proposed for assessing animal welfare. This paradigm primarily focuses on the promotion of positive states in an animal rather than eliminating all negative states [85,86,87]. In this model, the premise is that some negative experiences can never be completely eliminated in a captive environment but only temporarily counterbalanced and that they are essential for eliciting behaviors in the animal that are fundamental for their survival [85,86,87]. The assumptions are as follows: (a) Removing all negative experiences does not always result in good animal welfare; (b) The duration, intensity, and frequency of negative experiences must be within tolerable limits. The balance between positive and negative experiences will reflect the quality of life of that individual. This ultimately provides the animal with opportunities to choose positive experiences, thereby making their “lives worth living” [85]. Some of these biologically relevant experiences would include exploration of the habitat, food acquisition activities, and fish to fish interactions [85]. The working foundation of the model is that there are four separate domains (i.e., behavior, environmental, nutrition, and health) that culminate into a fifth domain (i.e., mental state) to provide an overall assessment of animal welfare. Therefore, animals that experience a net negative balance might have an affective state that includes anxiety, fear, panic, boredom, and depression, while animals that experience a net positive balance might feel contentment, pleasure, interest, confidence, and a sense of control [85,88].

Whether using the three outcome-based assessment or the Five Domains assessment, the best approach for evaluating fish welfare in most situations is to use a combination of these basic criteria. A list of criteria to be observed on a regular schedule should be established and modified for each species or population as necessary, and should include both diurnal and nocturnal observations. Fish in interactive exhibits should also be monitored in a similar manner for negative indicators but on a more abbreviated schedule. As mentioned previously, it is generally easier to recognize a fish in a negative welfare state. This can be accomplished by monitoring water quality parameters, observing fish for abnormal behaviors, and observing fish for lesions, injuries, or other clinical signs of disease. Either model should also take into consideration a grading system for the severity of the observed abnormality, such that a fish missing a few scales would not be considered in as serious a negative welfare state as a fish with a hemorrhagic, ulcerative skin lesion [12]. Thus, the ability to recognize the normal behavior and appearance of the fish or population is crucial to identifying both negative and positive indicators of welfare.

## 10. Conclusions

Aquariums and zoos are in a unique position to increase the understanding required to maintain and display a diverse variety of fish species in a positive state of wellbeing [89,90,91]. Habitat design, life support systems, water quality, nutrition, temperature, lighting, structures and substrates, and enrichment should provide a stimulating environment for the fish and promote natural species-specific behaviors [92]. This should include social and behavioral interactions between individuals of the same species and between different species in the community environment. Regular welfare assessment and review of assessment criteria is critical for advancing positive welfare in fish, and should be part of the standard routine during animal husbandry activities. Behavioral enrichment should provide species-appropriate challenges and opportunities so that it is an overall rewarding positive experience for the fish. A standard set of guidelines and procedures along with best practices should be developed for all individual aquatic species maintained in captivity [93,94]. These documents should also have specific sections detailing welfare evaluation and assessment standards that can be used to provide the best possible life for these animals. Ultimately the sharing of information between aquariums and zoological collections on both the negative and positive fish welfare experiences will advance both the study of fish welfare and the establishment of good welfare for fish in captivity.

## Figures and Tables

**Table 1:** Fish welfare evaluation using the three outcome-based assessment categories with indicators of each. A grading system for the severity of each observed abnormality should also be taken into consideration for an overall welfare assessment of the fish or population of fish.

Behavioral Indicators	Functional Indicators	Physiological Indicators
aggressive/submissive behavior	a. physical abnormalities	-hematology parameters
food intake/foraging	-buoyancy problems	-biochemistry parameters
schooling/shoaling behavior	-increased mucus production	-plasma cortisol levels
individual swimming behavior	-weight loss	-humoral antibodies
group swimming behavior	-increased respiratory rate	-heat shock proteins
abnormal behavior	-body abnormalities	-sex steroid levels
stereotypic/maladaptive behavior	-skin, fin and eye lesions	-expression of various genes
species-specific social behavioral	-changes in coloration	
lack of social display behavior	b. production parameters	
	-decreased growth rates	
	-decreased reproduction	
	-decreased egg production	
	-decreased hatching rate	
	-low survival of juvenile fish	
	-reduced growth	

## Data Availability

Not applicable.
